# Robotic Colorectal Cancer Surgery. How to Reach Expertise? A Single Surgeon-Experience

**DOI:** 10.3390/jpm11070621

**Published:** 2021-06-30

**Authors:** Michele Manigrasso, Sara Vertaldi, Pietro Anoldo, Anna D’Amore, Alessandra Marello, Carmen Sorrentino, Alessia Chini, Salvatore Aprea, Salvatore D’Angelo, Nicola D’Alesio, Mario Musella, Antonio Vitiello, Giovanni Domenico De Palma, Marco Milone

**Affiliations:** 1 Department of Advanced Biomedical Sciences, University of Naples “Federico II”, Via Pansini 5, 80131 Naples, Italy; mario.musella@unina.it (M.M.); antoniovitiello_@hotmail.it (A.V.); 2 Department of Clinical Medicine and Surgery, University of Naples “Federico II”, Via Pansini 5, 80131 Naples, Italy; vertaldisara@gmail.com (S.V.); pietro.anoldo@gmail.com (P.A.); anna.damore1993@libero.it (A.D.); alessandramarello@gmail.com (A.M.); carmensor94@gmail.com (C.S.); dr.alessiachini@gmail.com (A.C.); giovanni.depalma@unina.it (G.D.D.P.); milone.marco.md@gmail.com (M.M.); 3 “Federico II” University Hospital, Via Pansini 5, 80131 Naples, Italy; sa.aprea@gmail.com (S.A.); salvatore220987@hotmail.it (S.D.); nicodale1987@hotmail.it (N.D.)

**Keywords:** robotic, colorectal, colorectal cancer, laparoscopic, learning curve

## Abstract

The complexity associated with laparoscopic colorectal surgery requires several skills to overcome the technical difficulties related to this procedure. To overcome the technical challenges of laparoscopic surgery, a robotic approach has been introduced. Our study reports the surgical outcomes obtained by the transition from laparoscopic to robotic approach in colorectal cancer surgery to establish in which type of approach the proficiency is easier to reach. Data about the first consecutive 15 laparoscopic and the first 15 consecutive robotic cases are extracted, adopting as a comparator of proficiency the last 15 laparoscopic colorectal resections for cancer. The variables studied are operative time, number of harvested nodes, conversion rate, postoperative complications, recovery outcomes. Our analysis includes 15 patients per group. Our results show that operative time is significantly longer in the first 15 laparoscopic cases (*p* = 0.001). A significantly lower number of harvested nodes was retrieved in the first 15 laparoscopic cases (*p* = 0.003). Clavien Dindo I complication rate was higher in the first laparoscopic group, but without a significant difference among the three groups (*p* = 0.09). Our results show that the surgeon needed no apparent learning curve to reach their laparoscopic standards. However, further multicentric prospective studies are needed to confirm this conclusion.

## 1. Introduction

Minimally invasive surgery represents nowadays the standard approach for the treatment of colorectal pathologies [1,2,3]. However, the complexity associated with laparoscopic colorectal surgery requires several skills to overcome the technical difficulties related to this type of procedure [4,5]. Thus, the safety and the feasibility of laparoscopic colorectal surgery are related to the surgeon’s experience.

In this setting, several parameters have been investigated to define an adequate level of proficiency, but the number of cases needed to complete the learning curve is still not well defined [6,7,8,9,10,11], varying between 11 and 152 [6,9,11,12].

Recently, to overcome the technical challenges of laparoscopic surgery, a robotic approach has been introduced [13,14,15]. Its adoption to colorectal surgery has gained large consensus among the surgeons, because of its several facilities to overcome the difficulties of laparoscopic surgery.

In the setting of surgical expertise, most studies reported that robotic colorectal surgery has a shorter learning curve, reaching the plateau after 15–25 cases [12,16].

However, the results of the comparison between robotic and laparoscopic colorectal surgery during the learning curve are still under debate. This study reports the surgical outcomes obtained by the transition from laparoscopic to robotic approach in colorectal cancer surgery to establish in which type of approach the proficiency is easier to reach.

## 2. Materials and Methods

After the University Institutional Review Board of a tertiary referral colorectal center approval, a retrospective chart review of the minimally invasive colorectal resection for cancer performed by a single surgeon (M.M.) between 1 January 2014 and 31 March 2021 was conducted.

Patients who underwent colorectal resection for benign conditions and emergency cases were excluded.

Data about the first consecutive 15 laparoscopic (Group A) and the first 15 consecutive robotic cases (Group B) were extracted, adopting as a comparator of proficiency data about the last 15 laparoscopic colorectal cancer resections (Group C). Specifically, laparoscopic colorectal cancer surgery was introduced in our institution in 2014, while robotic-assisted surgery was adopted in 2018. Thus, the enrolment period ranged from 2014 (first 15 laparoscopic cases) to 2021 (last 15 laparoscopic cases) throughout 2018 (first 15 robotic cases).

### 2.1. Surgical Technique and Perioperative Management

When laparoscopy was introduced in the institution, the surgeon had no experience as the first surgeon in colorectal procedures, as well as for the robotic colorectal procedures when the robotic platform was introduced. 

All the patients underwent preoperative antibiotics and heparin prophylaxis as described previously [17].

All the surgical interventions were performed by the same surgeon (M.M.), who had no experience as the first surgeon in laparoscopic colorectal procedures at the beginning of the enrolment period. Similarly, the surgeon had no experience in robotic colorectal procedures at the enrolment of the first 15 robotic cases, but with adequate expertise in laparoscopic colorectal surgery.

All the patients underwent surgical procedures were under general anesthesia. In right colectomy, after identifying the ileocolic pedicle, the peritoneum was dissected towards the transverse colon, and the Toldt’s fascia was separated by the Gerota’s plane, preserving the duodenum, the right gonadal vessels, and the right ureter. After the ligation of the ileocolic pedicles, the right colic artery (if present), and the right branch of the middle colic artery, the right hemicolectomy was performed with a linear stapler (or with a robotic stapler during the robotic procedure), and an intracorporeal side-to-side isoperistaltic anastomosis was performed. In the left colectomy, after a coloepiploic detachment, the splenic flexure was completely mobilized by creating a window under the Inferior Mesenteric Vein (IMV) to separate the mesocolon from the pancreatic tail. After identifying the Inferior Mesenteric Artery (IMA) origin, the Toldt’s fascia was completely separated by the retroperitoneal plane, preserving the left ureter and the gonadal vessels. Then the IMV and IMA were ligated at their origin, a left hemicolectomy was performed, and an end-to-end Knight Griffen colorectal anastomosis was performed. In the case of splenic flexure resection, the transverse and descending colon were completely mobilized, the left branch of the middle colic artery and the left colic artery were ligated at their origin, and a splenic flexure resection was performed. In the case of transverse colon resection, both colic flexures were mobilized, and the wedge resection of the transverse colon and the mesentery between the two branches of the middle colic artery was performed. In the case of segmental colonic resection, an intracorporeal, isoperistaltic, side-to-side anastomosis was performed. In rectal anterior resection the procedure followed the surgical steps of the left hemicolectomy. In addition, a complete TME was performed, and in the case of middle- and low-rectal cancers, a protective loop ileostomy was performed after the Knight–Griffen colorectal anastomosis.

The postoperative period has been homogenized according to ERAS protocol [18].

### 2.2. Outcomes and Data Collection

Collected data of the three cohorts included gender, age, Body Mass Index (BMI), American Society of Anesthesiologists risk class (ASA), Charlson Comorbidity Index (CCI), tumor localization, and TNM stage, type of resection.

Intraoperative outcomes to predict the feasibility of the surgical approach were: Operative time, number of harvested nodes, and conversion rate.

Postoperative complications were recorded according to Clavien–Dindo (CD) classification [19], such as nausea and vomit, postoperative pain, ileus, surgical wound complications, abdominal or bowel bleeding, anastomotic leakage, need of Intensive Care Unit (ICU), and death.

Postoperative recovery outcomes were evaluated in terms of time to first flatus, time to first stools, and length of hospital stay.

The term anastomotic leakage included all conditions with clinical or radiologic anastomotic dehiscence, with or without needing surgical revision. Any bleeding has been considered if required blood transfusions. The term postoperative pain included the situations in which extra analgesia was needed for moderate or severe pain in the postoperative period. The term ileus included the situations in which the bowel movements were absent for over 72 postoperative hours. If the condition required prokinetics, it was inserted in the group of Clavien Dindo I complications group; on the contrary, the ileus requiring the insertion of the nasogastric tube was inserted in the Clavien Dindo II complications group. The term conversion included all situations in which a laparotomy was needed or in which, during the procedure, an extracorporeal anastomosis was preferred.

### 2.3. Statistical Analysis

Statistical analysis was performed by using SPSS version 26.0 (IBM, Armonk, NY, USA). Continuous data were expressed as mean ± SD; categorical variables were expressed as %. Continuous variables were compared among the groups by ANOVA test, and a Bonferroni post-hoc analysis was performed to investigate group differences on multiple dependent variables in the case of significance; categorical variables are compared by the *χ*2 test; when the minimum expected value was <5, the Fisher’s exact test was adopted. A *p* value of <0.05 was defined as statistically significant.

A subgroup analysis of the intraoperative outcomes was performed according to the tumor localization and consequent surgical procedures to exclude any bias-related to any surgical challenge.

## 3. Results

Our analysis included 45 patients, 15 in each group. Demographic data are reported in Table 1.

No significant difference was found among the three groups in terms of gender (*p* = 0.765), age (*p* = 0.814), BMI (*p* = 0.900), ASA Score (*p* = 0.557), Charlson Score (*p* = 0.978), tumor localization (*p* = 0.776), TNM (*p* = 0.946, *p* = 0.497 and *p* = 1.000, respectively) and type of surgical resection (*p* = 0.739).

Intraoperative outcomes, postoperative complications, and recovery outcomes are shown in Table 2.

Operative time was significantly longer in the first 15 laparoscopic cases (*p* = 0.001), and the Bonferroni post-hoc test confirmed this significance between Group A and both Group B and Group C (*p* = 0.003 and *p* = 0.008, respectively), while no significance was found between Group B and Group C (*p* = 0.998).

Similarly, a significantly lower number of harvested nodes was retrieved in the first 15 laparoscopic cases (*p* = 0.003). Bonferroni post-hoc test confirmed that the number of harvested nodes was significantly lower in Group A over both Group B and Group C (*p* = 0.003 and *p* = 0.047, respectively), while no significance was present between Group B and Group C (*p* = 0.944).

The number of conversions was similar among the three groups (*p* = 0.593).

Clavien Dindo I complications were seven in Group A, two in Group B, and three in Group C, showing no differences among the three groups, but a trend toward significance (*p* = 0.09). Ileus was included in the Clavien Dindo I complications because it did not require the nasogastric tube insertion. In a one-to-one comparison between Group A and Group B, the significance was present (*p* = 0.04).

Clavien Dindo II complications were 4 in Group A and 1 in Group B and C, respectively, with no significant differences among the three groups (*p* = 0.18).

Similarly, Clavien Dindo III complications were 1 in both Group A and Group B, and 0 in Group C, with no significant differences among the groups (*p* = 0.59).

No patients were affected by postoperative CD IV complications, and no death (CD V) occurred.

The comparisons between the overall complication rate showed a significantly lower number of complications in the first 15 robotic cases (*p* = 0.003).

About recovery outcomes, no differences among the three groups were found in terms of time to first flatus (*p* = 0.704) and time to first stools (*p* = 0.945). Interestingly, robotic approach was associated with lower length of hospital stay, with a trend toward the significance (*p* = 0.072).

### Subgroup Analysis

The results of the subgroup analyses on right hemicolectomies, left hemicolectomies, and anterior rectal resections are shown in Table 3, Table 4 and Table 5, respectively.

In the case of right hemicolectomy, subgroup analyses confirmed the results obtained in the main analysis. In fact, no differences were found in terms of conversions (*p* = 0.287), Clavien Dindo II complications (*p* = 0.09), Clavien Dindo III complications (*p* = 0.56), time to first flatus (*p* = 0.666), time to first stool (*p* = 0.391) and length of hospital stay (*p* = 0.530) among the three groups. Similarly, according to the main analysis, in Group A a longer operative time (*p* < 0.0001), a lower number of harvested nodes (*p* = 0.006), and a higher number of minor complications (Clavien Dindo I, *p* = 0.02) were present.

In the case of left hemicolectomy, there was no differences in terms of conversions (*p* = 0.517), Clavien Dindo II and Clavien Dindo III complications (*p* = 0.52 and *p* = 0.23, respectively), time to first flatus and stool (*p* = 0.369 and 0.992, respectively), length of hospital stay (*p* = 0.216) among the three groups, while a longer operative time and a lower number of harvested nodes were present in Group A (*p* < 0.0001 and *p* = 0.022, respectively), confirming the results of the main analysis. Differently to the latter, no differences were found in terms of Clavien Dindo I complications (*p* = 0.23) among the three groups in the case of left hemicolectomy.

In the case of anterior rectal resection, our subgroup analysis confirmed the longer operative time in Group A (*p* < 0.0001) and the non-significance among the three groups in terms of Clavien Dindo II complications (*p* = 0.41), time to first flatus and stools (*p* = 0.812 and *p* = 0.638) and length of hospital stay (*p* = 0.110). Interestingly, no statistical difference was found in terms of number of harvested nodes (*p* = 0.729) and postoperative Clavien Dindo I complications (*p* = 0.09).

## 4. Discussion

A minimally invasive approach is nowadays considered the treatment of choice of colorectal malignancies, being associated with low postoperative comorbidities and short length of hospital stay [20,21,22,23].

Nevertheless, it is still adopted less than expected, because of the complex surgical skills that this approach requires [4,5]. Thus, it should be considered safe only in expert hands.

In this setting, the correct proficiency in laparoscopic colorectal surgery could be considered as completed when the predefined variables reach a steady state, and the outcomes are comparable with those in the current literature [24,25].

Currently, several parameters have been proposed to determine the adequate number of procedures to achieve adequate expertise, but no consensus has been still reached among the author, varying the number of procedures between 11 and 152 [6,7,9,12,26].

In recent years, robotic surgery has been introduced to overcome some challenging skills of conventional laparoscopic surgery.

In fact, the intrinsic facilities of the robotic platform, i.e., the three dimensional view for better visualization of the operative field and the EndoWrist^®^ for more accurate movements in narrow spaces, allow to be less invasive and to obtain lower conversions rate over laparoscopic surgery [27,28,29,30].

Because of these facilities, the learning curve in robotic colorectal seems to be easier to complete, needing 15–25 cases to reach the plateau [12,16].

Furthermore, the learning curve of robotic surgery seems to be shorter in experienced laparoscopists [31].

The latter could depend on the fact that minimally invasive procedures have been standardized, and the differences between laparoscopic and robotic surgery are only related to the adoption of different surgical instruments.

During the last years, the interest in the adequate learning curve between robotic and laparoscopic colorectal surgery has become fervent.

Recently, Park et al. [32] compared 89 robotic and 89 laparoscopic rectal resections for cancer, demonstrating that the learning curve for robotic low anterior rectal resection was the 44th case, while for the laparoscopic approach, the 41st case. The authors assessed that the learning curves were similar, with similar clinicopathologic outcomes in both procedures.

On the contrary, De Angelis et al. [33] compared results from 30 robotic right colectomies and 50 laparoscopic right colectomies performed by a surgical fellow novice in minimally invasive colorectal surgery. The authors obtained that 16 was the number of cases necessary to complete the learning curve in the robotic group, while 25 in the laparoscopic one, concluding that the robotic approach was associated with a faster learning curve than conventional laparoscopy.

Being the debate about the learning curve between robotic and laparoscopic colorectal surgery still open, we decided to perform a comparison between the first 15 robotic and first 15 laparoscopic colorectal resections of a single surgeon, adopting as a comparator the last 15 laparoscopic cases, thus after the completion of the learning curve.

Our results showed that the first 15 robotic cases are associated with better postoperative outcomes than the first 15 laparoscopic cases. First, the operative time was significantly lower in the robotic group (*p* = 0.003), with a similar rate of conversion (*p* = 0.593).

From an oncologic point of view, in all groups, an adequate number of harvested nodes was obtained (>12), but a significantly higher number in the robotic group (Group B) was obtained (*p* = 0.003). Then, considering the postoperative complications, in the first laparoscopic group (Group A), a higher number of minor complications occurred (7 vs. 2, *p* = 0.04).

However, the safety of both laparoscopic and robotic procedures was confirmed by the low rate of major complications (1 anastomotic leakage in each group and no death).

Finally, the number of overall complications was significantly lower in the robotic group (*p* = 0.003).

These results were confirmed in the subgroup analysis after dividing the patients in accordance with the different types of resection (right hemicolectomy, left hemicolectomy, rectal anterior resection).

In the case of rectal resection, our results differed from the results by Park et al. [32]. In fact, the subgroups analysis confirmed that the robotic approach was associated with better outcomes over laparoscopy.

In the study by Park et al., the operative time between the laparoscopic and robotic groups was similar (about 202 and 208 min, respectively), as well as the mean number of harvested nodes (about 17 and 16, respectively). In our subgroup’s analysis in the laparoscopic groups, the operative time was significantly longer (267 vs. 184 min, *p* < 0.0001), while the mean number of harvested nodes was similar. On the contrary, in the study by Park et al., five conversions occurred in the laparoscopic group, while 0 in the robotic. In our subgroup analysis, no conversions occurred in both subgroups.

Finally, our subgroup analysis differed from the study by Park et al. for the number of major postoperative complications (Clavien Dindo IV and V). In fact, Park et al. registered 11 major complications in the laparoscopic group, and 3 in the robotic group, while we had no major complications.

In the case of subgroup analysis on right colectomies, our results confirmed the results obtained in the main analysis. Furthermore, we can state that the robotic approach could be considered feasible (only one conversion in the robotic group was needed) and safe in terms of postoperative complications. In fact, only one minor complication occurred (ileus, Clavien Dindo I), while no major complications and deaths were registered. On the contrary, in the first laparoscopic group, seven minor complications occurred (Clavien Dindo I complications and two Clavien Dindo II complications), and one major complication (one anastomotic leakage).

Our results are in line with the results obtained by De Angelis et al. [33], in which no conversions were needed in the robotic group, and four minor complications occurred (CD I–II complications).

Interestingly, comparing the robotic group with the last 15 laparoscopic cases, no differences were found in all included outcomes.

The possible reason for these similarities was that the surgeon was already an expert in laparoscopic colorectal procedures.

However, it is important to underline that surgical training with the DaVinci XI^®^ simulator has been performed by the surgeon before starting to adopt the robotic platform in colorectal procedures.

Odermatt et al. [31] has investigated the proficiency in rectal robotic procedures in experienced laparoscopist. In fact, comparing two surgeons with different expertise in laparoscopic rectal surgery (206 vs. 88 cases), the authors demonstrated that surgeon A needed no apparent learning process to reach their laparoscopic standards.

According to the current literature, our results showed that the surgeon needed no apparent learning curve to reach their laparoscopic standards.

Thus, we can state that a robotic approach to colorectal surgery could be considered safe and feasible in the hands of an expert laparoscopist.

A major limitation of the study must be addressed. First, the results are related to a single surgeon’s experience and derived from a retrospective study. Then, the cohorts are very small, making the comparison powerless. For this reason, larger comparative studies are needed to give definitive conclusions.

Thus, further multicentric prospective studies with the involvement of different surgeons and larger cohorts and are needed to confirm the absence of a learning curve in robotic colorectal procedures in experienced laparoscopists.

## Figures and Tables

**Table 1:** Demographic data of the included patients.

Patients’ Characteristics	Group A (*n* = 15)	Group B (*n* = 15)	Group C (*n* = 15)	*p* Value
Gender				0.765
M	7 (46.7)	8 (53.3)	9 (6.2)	
F	8 (53.3)	7 (46.7)	6 (40.0)	
Age	72.07 ± 7.9	70.53 ± 13.51	69.53 ± 10.44	0.814
BMI	26.4 ± 4.2	25.8 ± 4.03	26.16 ± 2.04	0.900
ASA Score				0.557
I	0 (0)	0 (0)	1 (6.7)	
II	11 (73.3)	8 (53.3)	9 (60)	
III	4 (26.7)	6 (40.0)	5 (33.3)	
IV	0 (0)	1 (6.7)	0 (0)	
Charlson Score	6.07 ± 1.8	6.13 ± 2	6.2 ± 1.26	0.978
Tumour localization				0.776
Caecum	2 (13.3)	2 (13.3)	0 (0)	
Right Colon	2 (13.3)	3 (20.0)	4 (26.7)	
Hepatic flexure	1 (6.7)	0 (0)	1 (6.7)	
Transverse colon	0 (0)	0 (0)	1 (6.7)	
Splenic flexure	0 (0)	0 (0)	1 (6.7)	
Descending colon	4 (26.7)	3 (20)	2 (13.3)	
Sigma	1 (6.7)	0 (0)	1 (6.7)	
Rectum	5 (33.3)	7 (46.7)	5 (33.3)	
T Classification				0.946
1	1 (6.7)	2 (13.3)	1 (6.7)	
2	5 (33.3)	3 (20.0)	5 (33.3)	
3	6 (40.0)	8 (53.3)	7 (46.7)	
4	3 (20.0)	2 (13.3)	2 (13.3)	
N Classification				0.497
0	9 (60)	6 (40.0)	10 (66.7)	
1	6 (40.0)	8 (53.3)	4 (26.7)	
2	0 (0)	1 (6.7)	1 (6.7)	
M Classification				1.000
0	14 (93.3)	14 (93.3)	14 (93.3)	
1	1 (6.7)	1 (6.7)	1 (6.7)	
Type of resection				0.739
Right hemicolectomy	5 (33.3)	5 (33.3)	5 (33.3)	
Transverse resection	0 (0)	0 (0)	1 (6.7)	
Splenic flexure resection	0 (0)	0 (0)	1 (6.7)	
Left hemicolectomy	5 (33.3)	3 (20.0)	3 (20.0)	
Rectal anterior resection	5 (33.3)	6 (40.0)	5 (33.3)	
Abdomino-perineal resection	0 (0)	1 (6.7)	0 (0)	

Dichotomous variables are expressed by number and (percentage); continuous variables by mean ± standard deviation. M: male; F: female; BMI: Body Mass Index; ASA: American Society of Anesthesiologists.

**Table 2:** Intraoperative outcomes, postoperative complications and recovery outcomes.

Outcomes	Group A (*n* = 15)	Group B (*n* = 15)	Group C (*n* = 15)	*p* Value
**Intraoperative outcomes**				
Operative time (min)	233 ± 55.48	169.66 ± 46.27	177 ± 42	**0.001**
Harvested nodes	17.73 ± 4.62	23 ± 3	21.53 ± 4.59	**0.003**
Conversion	1 (6.7)	1 (6.7)	0 (0)	0.593
**Postoperative complications**				
Clavien Dindo				
I	7 (46.7)	2 (13.3)	3 (20.0)	0.09
Nausea	4 (26.7)	1 (6.7)	1 (6.7)	
Ileus	3 (20.0)	1 (6.7)	2 (13.3)	
II	4 (26.7)	1 (6.7)	1 (6.7)	0.18
Wound infection	1 (6.7)	1 (6.7)	0 (0)	
Intraluminal bleeding	2 (13.3)	0 (0)	1 (6.7)	
Extraluminal bleeding	1 (6.7)	0 (0)4	0 (0)	
III	1 (6.7)	1 (6.7)	0 (0)	0.59
Anastomotic leakage	1 (6.7)	1 (6.7)	0 (0)	
IV	0 (0)	0 (0)	0 (0)	1.000
V	0 (0)	0 (0)	0 (0)	1.000
Overall complications	12 (80)	4 (26.6)	4 (26.6)	**0.003**
**Recovery outcomes**				
Time to first flatus (hrs)	56.53 ± 23.08	51.66 ± 19	49.47 ± 27.15	0.704
Time to first stool (hrs)	79.6 ± 21.06	77.47 ± 25.31	76.6 ± 28.71	0.945
Length of hospital stay (days)	4.5 ± 0.7	3.75 ± 0.93	4.34 ± 1.1	0.072

Dichotomous variables are expressed by number and (percentage); continuous variables by mean ± standard deviation. Hrs: hours.

**Table 3:** Intraoperative outcomes, postoperative complications and recovery outcomes on right hemicolectomies.

Outcomes	Group A (*n* = 15)	Group B (*n* = 15)	Group C (*n* = 15)	*p* Value
**Intraoperative outcomes**				
Operative time (min)	167 ± 4.47	114 ± 6.51	129 ± 6.51	**<0.0001**
Harvested nodes	16 ± 4.06	24.8 ± 2.1	21.2 ± 3.96	**0.006**
Conversion	2 (40)	1 (20)	0 (0)	0.287
**Postoperative complications**				
Clavien Dindo				
I	4 (80)	1 (20)	1 (20)	**0.02**
Nausea	2 (40)	0 (0)	0 (0)	
Ileus	2 (40)	1 (20)	0 (0)	
II	3 (60)	0 (0)	1 (20)	0.09
Wound infection	1 (20)	0 (0)	0 (0)	
Intraluminal bleeding	1 (20)	0 (0)	1 (20)	
Extraluminal bleeding	1 (20)	0 (0)	0 (0)	
III	1 (20)	0 (0)	0 (0)	0.56
Anastomotic leakage	1 (20)	0 (0)	0 (0)	
IV	0 (0)	0 (0)	0 (0)	1.000
V	0 (0)	0 (0)	0 (0)	1.000
**Recovery outcomes**				
Time to first flatus (hrs)	68.8 ± 29.31	64.2 ± 27.32	54 ± 21	0.666
Time to first stool (hrs)	93.6 ± 21.46	93.2 ± 32.73	74.4 ± 15.64	0.391
Length of hospital stay (days)	4.8 ± 0.83	4.3 ± 1.37	4.1 ± 0.57	0.530

Dichotomous variables are expressed by number and (percentage); continuous variables by mean ± standard deviation. Hrs: hours.

**Table 4:** Intraoperative outcomes, postoperative complications and recovery outcomes on left hemicolectomies.

Outcomes	Group A (*n* = 15)	Group B (*n* = 15)	Group C (*n* = 15)	*p* Value
**Intraoperative outcomes**				
Operative time (min)	235 ± 10	160 ± 13.23	193.33 ± 20.20	**<0.0001**
Harvested nodes	16 ± 2.45	22.66 ± 1.15	19 ± 3.63	**0.022**
Conversion	1 (20)	0 (0)	0 (0)	0.517
**Postoperative complications**				
Clavien Dindo				
I	2 (40)	0 (0)	0 (0)	**0.02**
Nausea	1 (20)	0 (0)	0 (0)	
Ileus	1 (20)	0 (0)	0 (0)	
II	1 (20)	0 (0)	0 (0)	0.52
Wound infection	0 (0)	0 (0)	0 (0)	
Intraluminal bleeding	0 (0)	0 (0)	0 (0)	
Extraluminal bleeding	0 (0)	0 (0)	0 (0)	
III	1 (20)	0 (0)	0 (0)	0.23
Anastomotic leakage	0 (0)	1 (33.3)	0 (0)	
IV	0 (0)	0 (0)	0 (0)	1.000
V	0 (0)	0 (0)	0 (0)	1.000
**Recovery outcomes**				
Time to first flatus (hrs)	49 ± 27.05	46.66 ± 8.08	38 ± 6.9	0.369
Time to first stool (hrs)	73.6 ± 22.74	72 ± 24	74.33 ± 20.59	0.992
Length of hospital stay (days)	4.5 ± 0.73	3.5 ± 0.8	4.11 ± 0.76	0.216

Dichotomous variables are expressed by number and (percentage); continuous variables by mean ± standard deviation. Hrs: hours.

**Table 5:** Intraoperative outcomes, postoperative complications and recovery outcomes on rectal anterior resection.

Outcomes	Group A (*n* = 5)	Group B (*n* = 6)	Group C (*n* = 5)	*p* Value
**Intraoperative outcomes**				
Operative time (mins)	297 ± 9	214 ± 6	221 ± 10	**<0.0001**
Harvested nodes	21.2 ± 5.44	21.5 ± 3.62	23.6 ± 6.42	0.729
Conversion	0 (0)	0 (0)	0 (0)	1.000
**Postoperative complications**				
Clavien Dindo				
I	2 (40)	0 (0)	2 (40)	0.09
Nausea	1 (20)	0 (0)	0 (0)	
Ileus	1 (20)	0 (0)	2 (40)	
II	0 (0)	0 (0)	1 (20)	0.41
Wound infection	0 (0)	0 (0)	0 (0)	
Intraluminal bleeding	0 (0)	0 (0)	0 (0)	
Extraluminal bleeding	0 (0)	1 (16.66)	0 (0)	
III	0 (0)	0 (0)	0 (0)	1.000
IV	0 (0)	0 (0)	0 (0)	1.000
V	0 (0)	0 (0)	0 (0)	1.000
**Recovery outcomes**				
Time to first flatus (hrs)	51.8 ± 10.26	44.33 ± 12.16	52.8 ± 39.43	0.812
Time to first stool (hrs)	71.6 ± 14.31	67 ± 18.14	83.4 ± 45.24	0.638
Length of hospital stay (days)	4.23 ± 0.56	3.46 ± 0.5	4.9 ± 1.7	0.110

Dichotomous variables are expressed by number and (percentage); continuous variables by mean ± standard deviation. Hrs: hours.

## Data Availability

Data are available by request to the corresponding author.
